# Therapeutic Properties of Edible Mushrooms and Herbal Teas in Gut Microbiota Modulation

**DOI:** 10.3390/microorganisms9061262

**Published:** 2021-06-10

**Authors:** Emanuel Vamanu, Laura Dorina Dinu, Diana Roxana Pelinescu, Florentina Gatea

**Affiliations:** 1 Faculty of Biotechnology, University of Agronomic Science and Veterinary Medicine, 59 Marasti Blvd, 1 District, 011464 Bucharest, Romania; laura.dinu@biotehnologii.usamv.ro; 2 Department of Genetics, University of Bucharest, 36-46 Bd. M. Kogalniceanu, 5th District, 050107 Bucharest, Romania; diana.pelinescu@bio.unibuc.ro; 3 Centre of Bioanalysis, National Institute for Biological Sciences, 296 Spl. Independentei, 060031 Bucharest, Romania; florentina.gatea@incdsb.ro

**Keywords:** pattern, miRNAs, antioxidant, SCFAs, polyphenols

## Abstract

Edible mushrooms are functional foods and valuable but less exploited sources of biologically active compounds. Herbal teas are a range of products widely used due to the therapeutic properties that have been demonstrated by traditional medicine and a supplement in conventional therapies. Their interaction with the human microbiota is an aspect that must be researched, the therapeutic properties depending on the interaction with the microbiota and the consequent fermentative activity. Modulation processes result from the activity of, for example, phenolic acids, which are a major component and which have already demonstrated activity in combating oxidative stress. The aim of this mini-review is to highlight the essential aspects of modulating the microbiota using edible mushrooms and herbal teas. Although the phenolic pattern is different for edible mushrooms and herbal teas, certain non-phenolic compounds (polysaccharides and/or caffeine) are important in alleviating chronic diseases. These specific functional compounds have modulatory properties against oxidative stress, demonstrating health-beneficial effects in vitro and/or In vivo. Moreover, recent advances in improving human health via gut microbiota are presented. Plant-derived miRNAs from mushrooms and herbal teas were highlighted as a potential strategy for new therapeutic effects.

## 1. Introduction

There are numerous published results on herbal products’ in vitro activity and edible or/and medicinal mushrooms. Only a small part of them are supported by an in vivo activity. The bioavailability of the target compounds is based on their molecular size and the solubilization capacity of the lipid carriers [1]. The result is low absorption, poor bioavailability, and the relatively small amount available to the body for physiological activity [2]. A well-known example in this regard is curcumin, present in *Curcuma longa*. It has low absorption, and in vitro studies have shown a modulatory activity on the microbiome by stimulating microbiota that generates butyric and propionic acid [3]. To be biologically active, herbal products must contain compounds that can be absorbed and regulate biological activity in a concentration-dependent manner [4]. The evaluation of the therapeutic potential of herbal teas takes into account the bioavailability of the main components. Their concentration is relatively low in teas and has only an adjuvant effect in classical therapies [5]. This aspect is less noticeable for mushrooms, as they also contain other classes of compounds that have a modulating effect, for example, polysaccharides and fibers [6]. Microbial dysbiosis is often associated with chronic pathologies such as type 2 diabetes and hypertension [7]. Fungi are also implicated in microbial modulation associated with attenuation or reversal of these chronic pathologies [7]. In both mushrooms and herbal teas, the determination of compounds with therapeutic impact remains a critical detail necessary for the characterization of the final product. Seasonal and geographical variability of these products plays a significant role in determining the quality of the product. Still, the effects on human health ensue from the characteristics of species [8]. These aspects influence the functional properties and lack of public awareness of these issues, leading to distrust in their use. Although they are not the only sources of functional compounds, mushrooms and herbal teas are some of the most used substrates in functional products. In addition, coffee or wine and the by-products resulting from their use (coffee grounds or leftovers from wine production) are alternative sources functional compounds that can be used to formulate innovative products [9].

Studies regarding human and animal gut microbiota biodiversity and dynamics have expanded during the last two decades, proving the essential role it plays in maintaining body host health. Implicitly, new research directions have been opened, aiming to determine the effect of the nutrients, food supplements, drugs, products belonging to non-conventional medicine, etc., on the microbiota structure. The diverse microbial strains are part of the human gut. They have beneficial effects on human health, including involvement in host metabolism, promoting immunity (innate and adaptative response) and defense against pathogens, synthesis of some essential nutrients, disease prevention, or reduced Firmicutes/Bacteroidetes ratio [10]. According to data obtained in international projects like the Human Microbiome Project and MetaHIT (Metagenomics of Human Intestinal Tract) [11], the number of microorganisms in the human gastrointestinal tract (GI) exceeds 10^10^. It includes bacteria, archaea and eukaryotes. The new sequencing technologies and bioinformatics software allowed the identification of 2172 species in the human GI microbiome classified into 12 different phyla, 93.5% of Proteobacteria, Firmicutes, Actinobacteria, and Bacteroidetes [12]. The dominant phyla are Firmicutes and Bacteroides. Some of the identified phyla include Verrucomicrobia, for which only one species, namely *Akkermansia muciniphila*, has been isolated from human GI [13,14,15].

In contrast to the host genome, the microbiome is characterized by high plasticity and the ability to adapt to various conditions. Thus, bacteria can break down polysaccharides that reach the colon to generate short chain fatty acids (SCFAs), that cross feed the microbiota and have physiological effects on gut integrity. Furthermore, the dynamics of GI microbiome composition are associated with many factors, including age, nutrition, health status [16], drug treatments [17], and the community [18].

Although fungi and herbal teas show a wide variety and profile of chemical constituents, several compounds are found in both. An example is rosemary acid, which has multiple therapeutic qualities and is a characteristic compound for *Boraginaceae* and *Lamiaceae* families of herbs, with the highest levels found in the *Nepetoideae* [19]. Given the varying concentrations in the different species, bioavailability has also been observed to vary between species [20]. The presence of rosemary acid in mushrooms has also been demonstrated in freeze-dried extracts from *Boletus edulis* [21]. An in vitro study showed that it is not stable, being degraded by transit through the stomach and small intestine. The stability of the bioactive component plays an important role in its antioxidant effect and ability to modulate the human gut microbiota [22]. Thus, the purpose of this mini-review is to highlight the essential aspects of modulating the microbial pattern by edible mushrooms and herbal teas. The phytochemical profile will be highlighted, and the phenolic compound’s action will be evaluated as the starting point of the modulating function. A new approach based on the increasing evidence that plant miRNAs might act as new bioactive compounds that directly modulate the gut microbiota is also investigated.

## 2. Interaction of Mushrooms with Human Microbiota

Edible mushrooms have been part of the human diet for thousands of years, but their beneficial effect in maintaining the consumers’ health has been studied only during the last decades has it been proved. Many mushrooms belonging to genera such as *Ganoderma*, *Pleurotus*, *Boletus*, *Inonotus*, *Grifola*, and *Armillaria* have been used for a long time as medicinal food due to their activities: antimicrobial, antiviral, antidiabetic, anti-inflammatory, hypocholesterolemic, and antitumor [23]. Lately, mushrooms are used as prebiotics and food supplements to improve consumers’ health [24].

Although the mechanisms involved in this beneficial effect are not yet elucidated, one hypothesis is the influence of the mushrooms on the GI microbiome composition. Mushrooms are rich in polysaccharides, proteins, vitamins, minerals, trace elements, and antioxidants. The in vitro and in vivo studies have shown the consequence of mushrooms consumption on the stimulation or depletion of some microbial phyla/species (Table 1).

As a functional food, edible mushrooms are a natural source of valuable compounds, modulating the microbiota pattern and metabolomic function. The use of mushroom species in microbiota modulation has resulted from the need to find new molecules with antimicrobial and anti-inflammatory activities. This need of the current biopharmaceutical industry results from the increase in the amount of antibiotics administered, which has led to the recurrent emergence of antibiotic resistance [30]. A related effect of this aspect is urinary tract infections with *Escherichia coli*, controlled using cranberry extracts [31]. The cranberry extract is the best known functional product for reducing and ameliorating the effects of recurrent *E. coli* infections.

Pathogenic strains support an inflammatory process in the human colon that leads to dysbiosis [32]. The impaired microbial balance increases oxidative stress by generating free radicals, which impact gut physiology and contribute to diseases such as colon cancer [33]. These phenomena negatively influence the whole body through the risk of developing various infections by decreasing immune function. In all these aspects, mushrooms are a functional product with multiple human body roles [34].

In addition to phenolic compounds, mushrooms also contain other functional components, such as β-glucans. β-glucans are polysaccharides made up of β-D-glucose units, which make up the cell-wall structure of mushrooms. These polymers are responsible for modulating the immune response, regulating blood sugar and reducing dietary cholesterol absorption [35]. β-glucans act as prebiotics and support the multiplication of favorable strains of the genus *Lactobacillus* [36]. One of the benefits is the microbiota-mediated activation of the immune system through T-cells and natural killer cells. Side effects are also mentioned and include flatulence or temporary drops in blood sugar [37]. In vitro studies have shown that β-glucans are recognized as receptors for immune system cells, such as macrophages, neutrophils, monocytes. In vivo, it has been demonstrated that the prebiotic effect is also generated by the polysaccharide structure that is not degraded by transit through the stomach and small intestine [38]. The fermentative breakdown of the molecule stimulates the nonspecific immune system, and this phenomenon is essential in the protection against tumor cell proliferation [39].

The prebiotic effect of edible mushrooms (especially wild edible mushrooms) may also be a modern strategy to prevent tumor cells’ proliferation by modulating the immune system [40]. The process may be related to modulation of the microbiota and reducing the proportion of bacteria that synthesize products with carcinogenic potential. Using a functional formula that modulates the metabolomic pattern is an effective alternative in the long-term protection of homeostasis [40,41]. For example, soluble dietary fibers from *Lentinula edodes* (LESDF-3) improved intestinal fermentation and increased the concentration of SCFAs, mainly propionic and butyric acid, and the number of *Bacteroides* sp. [42]. Another in vitro study showed that the use of β-glucans, present in the species *Pleurotus ostreatus*, *Pleurotus eryngii*, *Hericium erinaceus*, as a prebiotic, had a targeted action on the elderly by increasing cell number of. *Lactobacillus* spp. strains’ presence, and stimulated the synthesis of SCFAs, especially propionate and butyrate [28,43]. It can be considered that the fungi exert a species-specific action and can be included, as an adjuvant, in the nutritional plan of some target groups of the population. This aspect is influenced by many other exo- and endogenous factors, but the positive effect is observed in maintaining homeostasis in vulnerable groups of people.

The beneficial effects of mushroom consumption are given by the diversity of bio- active compounds, including polysaccharides, proteins, or secondary metabolites such as polyphenols, alkaloids, steroids, and terpenes [44]. The two types of polysaccharides present in fungi, homopolysaccharides, and heteropolysaccharides, can influence the microbiota depending on their structure and implicitly on their degree of solubility. The vast majority of polysaccharides in fungi are insoluble fibers (cellulose and lignin), and only a small part, β-D-glucans (such as lentinan from *Lentinus edodes*, schizophyllan from *Schizophyllum commune* or ganoderan from *Ganoderma lucidum*) are soluble compounds and are mainly responsible of the biological activity of fungi [45,46]. In the large intestine, various types of enzymes (hydrolases, esterases, lyases, transferases) are produced by microorganisms to metabolize polysaccharides in fungi. These enzymes are secreted mainly by Bacteroidetes but also by Firmicutes [47,48]. In support of these claims, numerous studies show that polysaccharides in fungi influence the increase in the number of specific bacterial strains and the decrease in others (Table 2).

## 3. Interaction of Microbiota with Herbal Teas

Due to the adverse side effects of drugs used in conventional medicine in the last century, there has been an increased interest in finding alternative solutions for different disorders prevention and treatment. Among the best-known alternatives are: probiotics, prebiotics, phytochemicals, and nutraceuticals. Herbal teas and edible mushrooms have a long history of use in certain health problems like heart diseases, diabetes, stomach conditions, liver disease, etc. [60,61].

Herbal teas are one of the most consumed beverages worldwide, both as a daily ritual and due to their numerous therapeutic properties [62]. Current global developments have led to an increase in the products available on the market, many of which are known only from the perspective of traditional medicine [63]. Interaction with the intestinal environment and bioavailability of key compounds are central in evaluating herbal teas’ modulatory function. Bioavailability depends on the type of tea, hot or cold, or how these drinks are consumed, both in volume and habit [5,64]. They contain functional compounds, which through an in vivo study, have been shown to improve certain physiological functions such as increased resistance to oxidative stress and synthesis of SCFAs [65]. Some examples of herbal tea and its beneficial effects are summarized in Table 3.

Polyphenols are the main bioactive compounds in teas. Polyphenols with monomeric and dimeric structures may be absorbed in the small intestine. However, most polyphenols, including complex polyphenols, oligomeric, and polymeric structures, reach the large intestine, metabolized by gut microbiota, or eliminated in the feces. Polyphenols reaching the large intestine can influence the diversity of microorganisms and regulate Firmicutes/Bacteroidetes ratio (Table 4).

In the large intestine, under the action of microbial enzymes, various reactions take place (C-ring cleavage, decarboxylation, dehydroxylation, and demethylation). Complex polyphenols are transformed into simpler compounds that are easily absorbed [71]. These reactions lead to the transformation of the initial polyphenols and the generation of intermediary products [72].

In vitro and in vivo studies mostly on animal models highlighted the inter-relationships between tea compounds and the GI microbiome. Data on the modulatory effect on GI microbiota by teas have been reported in several studies:

- Ginseng decoction—increased *Lactobacillus* spp. and *Bacteroides* spp. [86];
- Corn-starch tea—increased levels of Coriobacteriaceae, Lactobacillaceae, Prevotellaceae and Bifidobacteriaceae, and decreased Bacteroidaceae, Ruminococcaceae, Helicobacteraceae, and Enterobacteriaceae [87];
- Fuzhuan tea—an increase of *Lactobacillus* spp. [88];
- Green tea systematically modulates microbiota structure, depending on the original microbiota status and diet [85,86,87,88,89].

*Morus alba* leaf has been shown to stimulate *Bacteroides* and *Prevotella* in in vivo studies of farm animal gut microbiota. However, green tea inhibited the presence of potentially pathogenic strains [90]. This variation in microbial modulatory activity demonstrates the need for in vitro studies to examine the microbial proliferative activity of individual teas and their blends. Furthermore, this biological activity will be determined by the degree of bioavailability. The study is aimed exclusively at a group of target animals that could selectively consume this substrate. Another study with green tea showed a positive effect on HFD induced obesity [91]. These aspects show that regular consumption of tea can cause potentially ameliorate some of the effects of an HFD. What is unknown is the long-term effect, as it cannot be concluded that occasional consumption can cause a permanent change in the microbiota pattern. Inhibition of potentially pathogenic strains requires a long-term study to evaluate several aspects of functional compounds’ interaction with the microbiota [92].

An essential effect of green tea consumption has been demonstrated in vivo using pathogen-free male C57BL/6J mice at the age of 6–8 weeks. This study showed that tea consumption modifies microbiota and provides protection a HFD induced obesity. [91]. These data can be interpreted as a response that the microbiota has when drinking green tea. Microbial modulation results from regular consumption, dependent on the constant presence of biologically active compounds, such as catechins, epigallocatechin, or epicatechins [93]. The catabolism of these compounds was demonstrated by a previous in vitro study in rat microbiota [94].

Another essential aspect in recent years is the recovery of food waste, such as coffee or herbal tea [9,95]. The tea residues could be fermented, used as a feed additive for *Holstein heifers* (cattle) by the rumen’s anaerobic component, and caused an increase in immunoglobulins’ concentration and the general antioxidant status. This is a significant study that demonstrating beneficial effects throught the selective use of functional components, similar to the prebiotic-like impact [91]. In this regard, tea saponins are mentioned to stimulate the proportion of the genera *Bacteroides*, *Lactobacillus*, and *Bifidobacterium* strains. The saponins present in ginseng tea improve the Firmicutes/Bacteroidetes ratio, leading to the explanation of favorable effects on human health. The consumption of these plant species is associated [60,96]. The modulating role has also been demonstrated for black tea extract, which reduces physiological manifestations of colitis, regulates the TLR4/MyD88/NF-κB pathway, and causes a selective modulation of the microbiota in chemical-induced colitis in mice [97].

The relationship between microbiota and obesity is currently being studied extensively. Herbal teas are a group of products widely used in reducing obesity. Many formulas whose action is based on a diuretic effect only apparently solve the issue of weight gain caused by water accumulation [98]. Although water accumulation is a secondary cause of obesity, the anti-adipogenic effect is necessary to consider that a plant used to make tea is beneficial against obesity. In vitro study on mouse 3T3-L1 preadipocytes showed that hot water extract of *Chrysanthemum morifolium* Ramat flowers inhibited lipid accumulation. The activity of glycerol-3-phosphate dehydrogenase has led to an adipogenesis/lipogenesis-related gene expression and activation of the AMPK/SIRT1 pathway [99]. The lipid-lowering effect and cytotoxicities on human HepG-2 hepatocellular carcinoma cells have been demonstrated for ten novel dammarane-type saponins from a functional herbal tea made from *Gynostemma pentaphyllum* [100]. However, no direct link has been identified between the antilipidemic effect and the composition of these extracts. It is assumed that other mechanisms mediate the therapeutic effect. In this sense, the microbiota is expected to play a significant role because it interacts with saponins these molecules after consumption [101]. In vivo, the mechanism of action differs by being carried out by intermediate compounds, resulting from microbiota-mediated biotransformations. Microbiome dysbiosis can disturb the biotransformation process and determine the accumulation of adipocytes [102]. In vitro studies using newly isolated compounds on cell line models data on cell line efficiency tended to capitalize on this source of functional compounds in identifying alternative mechanisms for modulating human physiological functions in chronic obesity.

**Figure 1:**
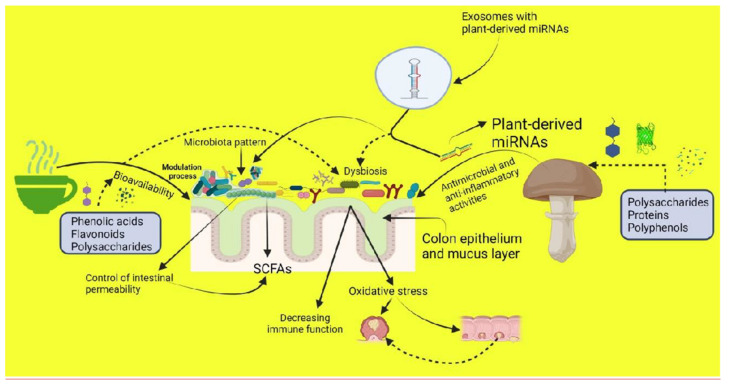
The action of herbal teas and mushrooms consumption effects on gut microbiota bioactivities. The figure was drawn using bioRender Premium Edition (https://app.biorender.com/; accessed on 10 May 2021).

Moreover, Diez-Sainz et al. hypothesize that specific gut microbes could control intestinal permeability by increasing/decreasing bioavailability and bioefficiency of plant bioactive compounds and their concentration in blood circulation for potential biological actions [103]. Supportive data showed that microbiota dysbiosis could disrupt the intestinal barrier and promote or help the progression of diverse pathologies (Figure 1). Changes in gut microbiota composition promote variability in the uptake of plant bioactive molecules [104,105].

The large majority of the scientific data reported show the beneficial effects of tea administration and mushroom consumption on the gut microbiota. Less data regarding their possible adverse effects were published: the presence of toxic compounds like aristocholic acid, which can have a negative impact on gut microbiota homeostasis [106]. Increasing cell number of some microbial strains like *Lactobacillus* spp. may sometimes induce interleukin 1 β, which may be implicated in aggravation of the inflammatory response [107]. Consumption of 400 mL of liquid green tea (LGT) for ten days by human volunteers led to an elevation of Firmicutes, reduction of Bacteroidetes, and elevation of the ratio of Firmicutes to Bacteroidetes in microbiota from feces. The consumption of LGT favored the increase of the number of bacteria such as *Lachnospiraceae*, *Ruminococcaceae*, and *Bifidobacteriaceae*. Those are responsible for the synthesis of SCFAs. These changes persisted even one week after LGT administration [108].

## 4. Effect of Plant miRNAs on Gut Microbiota Modulation with Impact on the Human Health

MicroRNAs (miRNAs) are a class of small intracellular single-stranded and non-coding RNAs from 18–25 nucleotides that play essential roles in gene expression, individually or in combination with other miRNAs. Thousands of miRNAs have been discovered in prokaryotes and eukaryotes, with more than 2500 miRNAs identified in humans [109]. In both plant and animal cells, microRNAs regulate gene expression at the posttranscriptional and posttranslational levels, mainly targeting messenger RNAs (mRNAs), resulting in mRNAs translation repression, degradation, or both. Thus, microRNAs regulate more than 60% of human protein-coding genes and play a crucial role in various biological processes. Simultaneously, deregulation of miRNAs disturbs key molecular events that are associated with different pathologies [110]. Interactive analysis showed that certain miRNAs species, such as miRNA-155, miRNA-168, miRNA-854 family, may be expressed in both plant and animal cells while miRNA-21, miRNA-146a, and miRNA-155 are coexisted in the gut microbiome and foods. Thus, dietary miRNAs may potentially contribute to cross-kingdom communication and modulate molecular mechanisms associated with human health and disease [111,112].

In recent years, many efforts to develop miRNAs-based therapeutics to treat human diseases were performed. Targeting natural compounds from plants, such as flavonoids, terpenoids, alkaloids that modulate human miRNAs, is an important strategy in cancer treatment [113,114]. Another approach is based on the increasing evidence that plant miRNAs might act as new bioactive compounds that directly or thoroughly the gut microbiota modulates health-associated miRNAs levels. However, there is a controversy if the exogenous plant miRNAs can penetrate the human bloodstream and reach a circulating level that allows them to act as bioactive ingredients [102], influence gut microbiome and positively affects the host health, acting as a cross-kingdom gene expression regulator raises attention. This hypothesis is based on the following rationale. First, the influence of plant-derived diet and medicinal plants on microbiota composition, which has been extensively investigated [103,115]. Second, gut microbiota and endogenous/exogenous miRNAs bidirectional interaction have been reported, as they can influence each other and regulate the host pathology [110,112]. Gut microbiota has been found to miRNA-regulate the host gene expression via the production of metabolites, such as lipopolysaccharide, butyrate and amyloids, and other signaling molecules. On the other hand, the host shapes and controls the gut microbiome by miRNAs secreted by the epithelial cells into the gut lumen, then found in fecal content, specifically targeting bacterial genes [110,112,116].

However, how edible plant or herbal medicine miRNAs could shape gut microbiota composition by modulating microbe genes that affect growth is poorly understood. It has recently been suggested that diet plant-derived miRNA-146a directly modulates the structure and composition of the gut microbial communities within 1–2 weeks. Still, the changes in microbial community structure were modest [117]. Another way plant-derived miRNAs could modulate gut microbiota with physiological consequences on the host is via extracellular vesicles, exosomes, and exosome-like nanoparticles (ELNs). Exosomes are one of the natural carriers of miRNAs that protects their integrity and stability and play essential roles in cell-to-cell communications. Teng et al. proved that ginger-derived ELNs could be selectively taken up by *Lactobacillus rhamnosus*. The diverse miRNAs carried inside that target bacterial genes could regulate the composition, metabolites, growth, and localization of the gut microbiota, finally improving colitis in mice [118]. This pioneering study established a direct causal relationship between plant miRNAs and gut microbiota. It proposed a therapeutically approaches based on manipulation of the microbiome with plant miRNAs for treatment of dysbiosis-related disease. Similar research provides strong evidence that dietary milk exosomes change the gut bacterial community’s composition in mice [119].

Medicinal herbal teas and mushrooms have been used to treat diseases for centuries. They contain thousands of miRNAs that might act as hidden bioactive ingredients in- volved in their therapeutic effects [120,121]. Medicinal plant-derived miRNAs’ stability during harsh conditions of preparation and storage is crucial for their therapeutic potential. It has recently been confirmed that some miRNAs survived during the herb preparation process [122]. However, the knowledge of plant medicinal-derived miRNAs in regulating human health via microbiome is at a very early, exploratory stage. Plant medicinal-derived miRNAs-based therapeutics is a new concept with a wide range of practical applications for human health and two important advantages, lower side effects and price. Still, more research must be done to obtain high-efficiency therapies to prevent or treat human diseases [123].

## 5. Conclusions

The way in which the bio-active compounds influence the gut microbiota in teas and mushrooms depends on a multitude of factors starting with their frequency of consumption, the compounds concentration, the pattern of the bioactive compounds and the health of the organism. Edible mushrooms and herbal teas exert beneficial effects on human health both directly and indirectly by the modulation of GI microbiome. It is not very clear whether plant medicinal-derived miRNAs affect the gut microbiota modulation process. Still, it is thought that they may affect the plasticity of the human microbiome. Effect on cytotoxicity induced by oxidative stress or gut microbial populations is still a new direction that could imply mushrooms or herbal teas as functional products.

## Figures and Tables

**Table 1:** Impact of mushrooms consumption on gut microbiota pattern.

Mushrooms	Gut Bacteria Increased	Gut Bacteria Depleted	Human/ Animal	References
*Hericium erinaceus* (Lion’s mane mushroom)	*Bifidobacterium, Coprococcus, Desulfovibrio, Lactobacillus, Parabacteroides, Prevotella*	*Corynebacterium, Dorea, Roseburia, Ruminococcus, Staphylococcus, Sutterella*	Animal (rat)	[25]
*Ganoderma lucidum* (Reishi)	Firmicutes, Proteobacteria (*Helicobacter*), *Rikenella*	*Acinetobacter*, Actinobacteria (*Arthrobacter*, *Corynebacterium*), Bacteroidetes (*Bacteroides*, *Parabacteroides*, *Prevotella*), *Blautia*, *Brevundimonas*, *Clostridium*, *Coprobacillus*, *Cyanobacteria*, *Facklamia*, *Jeotgalicoccus*, *Sporosarcina*, *Staphylococcus*, *Streptococcus*	Animal (mice)	[26]
*Boletus edulis*, *Boletus pinophilus*, *Boletus aureus* (Porcini), *Armillaria mellea* (Honey fungus), *Lactarius piperatus* (blancaccio), *Pleurotus eryngii* (King oyster)	*Bifidobacterium* and *Lactobacillus* genera	-	Human	[27]
*Cyclocybe cylindracea* (poplar mushroom), *Hericium erinaceus*, *Pleurotus eryngii*, *Pleurotus ostreatus* (Oyster mushroom)	*Bifidobacterium* spp. *Faecalibacterium prausnitzii (Ruminococcaceae)*, *Eubacterium rectale/Roseburia* spp.	-	In vitro study using human faeces	[28]
*Flammulina velutipes* (Enoki), *Hypsizygus marmoreus*, (White beech mushroom), *Lentinula edodes* (Shiitake), *Grifola frondosa*, (Maitake) *Pleurotus eryngii*	*Allobaculum*, *Bifidobacterium*, *Ruminococcus*, *Lactobacillus*, *Lactococcus*, *Streptococcus*	*Bacteroides*, *Prevotella*, *Mucispirillum*, *Dorea*, *Roseburia*, *Anaerotruncus*, *Oscillospira*, *Escherichia* and *Akkermansia*	Animal (mice)	[29]

**Table 2:** Effect of polysaccharides from various mushrooms on gut microbiota.

Mushrooms (Common Name)	Bioactive Compounds	Type of Study	Gut Bacteria Effects	References
*Lentinula* *edodes* (Shiitake)	*Lentinula edodes* soluble dietary fiber fractions (LESDF): LESDF-1: →6)-β-D-Glcp-(1→,→4)-β-D-Glcp-(1→,→5)-β-D-Arap-(1→,→4)-β-D-Xylp-(1→,→4)-α-D-Manp,→3)-α-L-Rhap-(1→,→6)-β-D-Galp-(1→ LESDF-2: →6)-β-D-Glcp-1→,→4)-α-D-Glcp-(1→,β-DGlcp-(1→,→5)-β-D-Arap-(1→,→2)-α-L-Rhap-(1→,→3,6)-α-D-Manp-(1→,→6)-β-D-Galp-(1→,→4)-β-D-Xylp-(1→ LESDF-3: β-D-Arap-(1→,→3)-α-D-Galp-(1→,→3,6)-α-D-Manp-(1→, →4)- β-D-Xylp-(1→, and →2,4)-α-D-Glcp-(1→	Human gut microbiota in vitro study	LEDS-2 increase microbial communities LEDS-3 causes an increase in the abundance of *Parasutterella*, *Bacteroides*, *Parabacteroides* and *Lachnospira*	[42,49,50]
*Auricularia auricula-judae* (wood ear) *Flammulina velutipes* (velvet shank)	Mushrooms dried powder after in vitro digestion with α-amylase, pepsin, and pancreatin. The main bioactive compounds are carbohydrates and proteins.	Human gut microbiota in vitro study	They led to an increase in the abundance of groups of Actinobacteria, Bacteroidetes, Proteobacteria and inhibited the growth of Fusobacteria and Firmicutes	[51]
*Lentinus edodes Pleurotus eryngii* (King Oyster)			Promoted the growth of Bacteroidetes, Actinobacteria and inhibited the development of Proteobacteria, Fusobacteria, and Firmicutes	
*Pleurotus osteratus* (*Oyster mushroom*)			Promoted the growth of Actinobacteria, Bacteroidetes, and Fusobacteria, inhibited the growth of Proteobacteria and Firmicutes.	
*Agaricus bispours* (champigno n)			Positively influences Actinobacteria, Fusobacteria, and Firmicutes and inhibit the growth of Bacteroidetes and Proteobacteria	
*Hericium erinaceus* (lion’s mane mushroom)	Polysaccharides, alcoholic extracts, and whole extracts alcoholic extracts, and whole extracts	Rats with inflammatory bowel disease	Reduce the amount of lipopolysaccharide toxins, increase the abundance of *Bifidobacterium*;	[25,52]
*Oudemansiella* *radicata* (Rooted Collybia)	Polysaccharide extract	Human gut microbiota in vitro study	Reduce the Firmicutes/Bacteroidetes ratio Increase *Bacteroides* abundance	[53]
*Ophicordyceps sinensis* (Rooted Collybia)	Mushrooms dried powder after in vitro digestion with α-amylase, pepsin, and pancreatin	Human gut microbiota in vitro study	Increase abundance of *Bifidobacteriales*, *Selenomonadales*	[54]
*Cordyceps* *militaris* (Chinese caterpillar fungus)			Increase the relative abundance *Bacteroidales*. Implicitly decreasing the ratio of Firmicutes/Bacteroidetes ratio	
*Inonotus obliquus* (chaga)	Polysaccharides Ethanolic extract	High-fat diet mice (HFD-mice)	Increase *Akkermansia* abundance and fatty acid elongation	[55]
*Phellinus linteus* (black hoof mushroom)	Polysaccharide total extract (two fractions were characterized, PLPS-1: α-D-glucose (1→4)-α-D-glucose (1→6) units and PLPS-2: α-(1→3)- D-glucose and α-(1→6)-D-glucose)	Sprague Dawley rats with Type 2 diabetes	Causes an increase in the abundance of *Lachnospiraceae*-NK4A1 36, *Blautia*, *Ruminiclostridium*-9, *Eubacterium* *xylanophilum*, *Anaerotruncus*, *Oscillibacter Lachnospiraceae*-UCG-00 6, *Roseburia*, *Prevotella* and improves microbial balance	[56,57]
*Cordycepssinensis* (Cordyceps mushroom)	Polysaccharide fraction	HFD mice	The relative abundances of *Actinobacteria* (in particular *Olsenella* bacteria) and *Acidobacterias* were increased and those of Bacteroidetes decreased. At the genus/cluster level, decreases of *Barnesiella*, *Prevotellaceae* and the *Lachnospiraceae incertae sedis* and increases of *Christensenella*, *Clostridium*_XVIII cluster and *Pseudomonas*	[58]
*Pleurotus* *eryngii*	Soluble polysaccharide fraction	HFD mice	Supplementation causes changes only at the genus level, an increase of *Anaerostipes*, *Clostridium*, *Lactococcus*, and a decrease of *Roseburia* and *Lactobacillus*	[59]

**Table 3:** Most common herbal teas and health effects.

Herbals	Beneficial Effects	Reference
*Matricaria recutita* (Chamomile)	Anti-inflammatory, antispasmodic, antioxidative activity, antiplatelet activity	[66]
*Mentha piperita* (Peppermint)	Antimicrobial and antiviral activity, analgesic and anesthetic effects, immunomodulatory activity	[67]
*Cinnamomum zeylanicum* (Cinnamon)	Antimicrobials activity, anti-inflammatory, antiviral, antioxidant, antitumoral activity, Cholesterol- and Lipid-Lowering Properties	[68]
*Petroselinum crispum* (Parsley)	Antioxidant, hepatoprotective, anti-diabetic, analgesic, immunosuppressant, anti-platelet, gastroprotective, cytoprotective, laxative, estrogenic, diuretic, hypotensive, antibacterial, and antifungal activities	[69]
*Camellia sinensis* (Macha tea)	Antimicrobial activity, anti-inflammatory	[70]

**Table 4:** Tea bioactive compounds and their effects on gut microbiota.

Tea	Bioactive Compounds	Type of Study	Gut Bacteria Effects	References
Kudingcha (KDC) from *Ilex latifolia* Thun and *Ilex kudingcha* C.J. Tseng (large-leaved Kudingcha)	Neochlorogenic acid, chlorogenic acid, cryptochlorogenic acid, dicaffeoylquinic acids isomers, quercetin with different glycosides triterpenoid saponins, polysaccharides, monosaccharides, proteins, simple organic acid.	HFD mice	Administration of KDC led to a reduction in abundance of *Erysipelotrichaceae*.	[73,74,75]
Fuzhuanbrick Tea post-ferment ed tea (dark tea leaves of *Camellia sinensis* var. *sinensis* and *C. sinensis* var. *assamica*) (border-selling tea or border-tea)	Gallic acid, catechins, free amino acids, alkaloids and volatile components.		Administration of the tea reduces the Firmicutes/Bacteroidetes ratio and has led to the increased of relative abundance in *Bifidobacteriaceae*	
KDC and FBT			Led to an decreased of *Clostridium*, *Bilophila*, *Oscillibacter*, *Lactonifactor*, *Eisenbergiella*, *Olsenella*, *Leuconostoc*, *Pseudoflavonifractor* and *Streptococcus*.	
Green tea (*Camellia sinensis*)	Standardized green tea extract: catechins (49.9%), including epigallocatechin (9.7%), epicatechin (5.4%), epigallocatechin-gallate (28.4%), and epicatechin-gallate (6.4%), as well as caffeine (4.5%) and theanine (0.4%). Other possible biologically active compounds: gallic acid, *p*-coumaric acid and quinic acid derivatives, caffeoylquinic acid isomers, and caffeoyl, kaempferol 3-*O*-*p*-coumaroylglucoside and kaempferol 3-*O*-*p*-coumaroyldirhamnosylhexosi de	Mice under UV stress	7-day supplementation of green tea extract Firmicutes/Bacteroidetes ratio and increased levels of *Lactobacillus* spp. and *Bifidobacteriumspp*.	[76,77]
*Ligustrum* *robustum* (Roxb.) Blume (bora-bora, Ceylon privét, privet, tree privet, troene)	*Ligistrum robustum* ethanol extract (LRE)—glycosides extract. Ligupurpuroside, acteoside, isoacteoside, ligupurpuroside A, ligupurpuroside B, ligupurpuroside C, ligupurpuroside D, and osmanthuside B	HFD mice	After 16 weeks of LRE administration, the ratio of Firmicutes/Bacteroidetes ratio increased. LRC contributed to growth stimulation of belonging of *Streptococcus*, *Lactobacillus*, and *Eubacterium coprostanoligenes* groups and a *Coriobacteriaceae*_U CG-002, and *Lachnospiraceae* groups.	[78]
*Solidago virgaurea* L. (European goldenrod, Woundwort)	Solidago v. Infusion extract Mainly caffeoylquinic acid derivatives (caffeic acid, p-coumaric acid, chlorogenic acid, neochlorogenic acid, cryptochlorogenic acid) flavonoids (quercetin rhamnohexoside, rutin, isoquercetin, kaempferol) and some phenylpropanoids.	Human and swine gut microbiota in vitro study	In human and swine cultures gut microbiota takes place hydrolysis of caffeoylquinic acid derivatives and deglycosylation of flavonoids.	[79]
*Chrysanthem um morifolium* (florist’s daisy and hardy garden mum or juhua)	Hot-water extract. Chlorogenic acid, tuberonic acid glucoside, diglucosylapigenin isomer, naringenin-6,8-di-C-glucoside Isookanin-7-O-β-diglucopyranoside, Quercetin-3-O-galactoside, diglucosyapigenin isomer, luteolin-7-O-glucuronide, luteolin-7-O-glucoside, dicaffeoylquinic acid isomer, apigenin-7-O-rutinoside, apigenin-7-O-glucoside, Kaempferol-3-O-acetyl-glucoside, diosmetin 7-O-rutinoside, diosmetin 7-glucuronide, acacetin-7-O-6″-malonylgactoside, apigenin 7-O-acetylglucoside isomer, apigenin 7-O-acetylglucoside isomer, Ombuin-3β-rutinoside, luteolin, apigenin 7-O-acetylglucoside isomer acacetin-7-O-glucuronide, acacetin, apigenina, diosmetin, eupatorin, casticina.	Regular chow diet fed C57BL/6J mice study	Hot water extract administration has shaped gut microbiota by increasing Bacteroidetes, Firmicutes (*Prevotella*) *Bifidobacterium* 6J mice with gut Colonized by microbiota coming from healthy human volunteers.	[80]
*Cyclocarya* *paliurus* (Wheel Wingnut; sweet tea tree)	Water extract of *C. paliurus* leaves. *Cyclocarya paliurus* flavonoids: Kaempferol-3-O-β-glucuronide, kaempferol-3-O-α-L-rhamnopyranoside, isoquercitrin, quercetin	Adult male C57BL	The administration of CPF led to an increase of microbial diversity, reduction in the relative abundance of *Faecalibacterium*, *Mitsuokella*, *Ruminococcus*, *Desulfovibrio* and *Megamonas*.	[81]
*Edgeworthia* *gardneri* (papertree, paperbush; Argelee)	Water extract, phloroglucinol, swainonine, trigonelline, coumalic acid, Coumarin, scopolamine, 7,8-Dihydroxy-4-methylcoumarin, chlorogenic acid, berberine, psoralen, apigenin, caffeic acid, γ-Terpinene, rutin, 4-methylumbelliferone, scopoletin, kaempferol-3-O-rutinoside, α-Pinene, daidzein, bergapten, glycitein, cytosine, α-Linolenic acid, ferulic acid, palmitoleic acid, linoleic acid, stearic acid, trans-vaccenic acid, arachidonic acid.	HFD mice	The microbial diversity was improved. The extract decreases the number of Proteobacteria and Deferribacteres and reverses the levels of *Clostridiales*, *Lachnospiraceae*,S24–7, *Rikenellaceae*, and Dorea in diabetic mice.	[82]
*Salvia miltiorrhiza* Bge (red sage or Danshen)	Ethanolic extract, Danshensu, protocatechualdehyden, caffeic acid, rutin, isoquercitrin, astragalin, rosmarinic acid, lithospermic acid, salvianolic acid B, salvianolic acid A, and salvianolic acid C	C57BL/6J diabetic mice	Decrease of *Proteus hauseri* and *Helicobacter winghamensis* abundance and growth stimulation of *Anaerotruncus colihominis*, *Mucispirillum schaedleri*, and *Butyricimonas virosa*. The extract increases the biodiversity and species of the gut microbiota and reduces the Firmicutes/Bacteroidetes ratio.	[83]
*Hypericum attenuatum* Choisy (St. John’s Wort)	Ethanolic extract, rutin, Quercetin-3-O-β-D-glucuronide	Male KM diabetic mice	The extract reverses dysbiosis induced by diabet, increases levels of *Clostridiaceae*, *Erysipelotrichaceae* and *Lactobacillaceae*	[84]
Decaffeinated green tea (GT) and black tea (BT)	Ethanolic extracts of green tea (GTP) and black tea (BTP), gallic acid, epigallocatechin gallate, epicatechin gallate, epigallocatechin, epicatechin	Male mouse C57BL	6J mice (strain JAX 000664), low-fat/high sucros e diet (LF/HSD), HFD/HSD, HFD/HSD supplemented with GTP and BTP. HFD/HSD-GTP and BTP diets lead to a significant increase in the relative proportion of *Parabacteroides*, *Bacteroides*, and *Prevotella* and an increase of *Roseburia*, *Bryantella*, *Lactococcus*, *Lactobacillus*, *Blautia*, *Anaerostipes*, *Shuttleworthia*, and *Acetitomaculum*, *Collinsella*. GTP administration leads to an increase cell number of bacterial strains belonging to *Clostridium* and *Coprococcus* and a decrease of *Turicibacter* and *Marvinbryantia*. BTP consumption was increase in *Oscillibacter*, *Anaerotruncus*, and *Pseudobutyrivibrio*	[85]

## Data Availability

Not applicable.
